# Characterization, biological evaluation and molecular docking of mulberry fruit pectin

**DOI:** 10.1038/s41598-020-78086-8

**Published:** 2020-12-11

**Authors:** R. Venkatesh Kumar, Devika Srivastava, Vandana Singh, Umesh Kumar, Vijay Kumar Vishvakarma, Prashant Singh, Dinesh Kumar, Rajesh Kumar

**Affiliations:** 1 Department of Zoology, Babasaheb Bhimrao Ambedkar University, Vidya Vihar, Raibareli Road, Lucknow, 226025 India; 2 Department of Chemistry, Atma Ram Sanatan Dharma College, University of Delhi, New Delhi, 110021 India; 3 Centre of Biomedical Research, Uttar Pradesh, SGPGIMS Campus, Raebareli Road, Lucknow, 226014 India; 4 Department of Microbiology, Babasaheb Bhimrao Ambedkar University, Vidya Vihar, Raibareli Road, Lucknow, 226025 India

**Keywords:** Biological techniques, Cancer, Computational biology and bioinformatics, Microbiology

## Abstract

Contemplating the exemplary benefits of pectin on human health, we precisely characterized and evaluated the antibacterial and anticancer activities from purified Mulberry Fruit Pectins (MFP). Here, we tested BR-2 and S-13 varieties of mulberry fruit pectins against six bacterial strains and two human cancer cell lines (HT-29 and Hep G-2), using MIC and an in vitro cell-based assay respectively. The BR-2 mulberry fruit pectin performs superior to S-13 by inhibiting strong bacterial growth (MIC = 500–1000 μg/mL) against tested bacterial strains and cytotoxic activities at the lowest concentration (10 µg/ml) against the Hep G-2 cell line. However, both tested drugs failed to exhibit cytotoxicity on the human colon cancer cell line (HT-29). Based on molecular interaction through docking, pectin binds effectively with the receptors (1e3g, 3t0c, 5czz, 6j7l, 6v40, 5ibs, 5zsy, and 6ggb) and proven to be a promising antimicrobial and anti-cancer agents. The pursuit of unexploited drugs from mulberry fruit pectin will potentially combat against bacterial and cancer diseases. Finally, future perspectives of MFP for the treatment of many chronic diseases will help immensely due to their therapeutic properties.

## Introduction

Pectin is a carbohydrate polymer existing in almost all plants where it contributes to the cell structure as a cementing agent. Pectin comprises a number of polymers which differ according to their molecular weight, chemical configuration, and content of neutral sugars. The different plant produces pectin with diversified functional properties^1^. Pectin composition is affected by their origin, localization within the plant, and the extraction method used to obtain them. Pectin is a family of polysaccharide^2^ that contain 1,4-linked α-D-galactopyranosyluronic acid residues^3^ and these are consist of three major classes namely, homogalacturonan (HG), rhamnogalacturonan I (RG-I) and rhamnogalacturonan II (RG-II)^4^ and they have a wide range of applications including emulsifier, gelling agent, thickener, stabilizer, and fat or sugar replacer in low-calorie foods and also used as an essential ingredient in functional foods^5^. Pectins are natural sources that are exploited for the preparation of jam, jellies, and preservatives as they are demonstrated remarkable gelling properties^1^. Due to having gel-forming properties, the biomedical applications of pectin have been growing exponentially in the area of drug delivery, tissue engineering, and wound dressing. Recent studies witnessed the various biological activities of pectin, such as antioxidants, antitumor, anti-inflammatory^6^, and antibacterial activities as well^7^. Bacterial spp. Such as *Staphylococcus* spp. and *Streptococcus* spp. are responsible for the pathogenesis of respiratory and skin infections, whereas pseudomonads involve in gastrointestinal, urogenital diseases, and wound contamination. These species are resistant to almost all of the older antibiotics. Therefore, to treat such bacterial infectious diseases, the expansion and development of alternative drugs are the current priority^8^. In recent days, pectins are fetching great attention due to their antibacterial activity against Gram-positive and Gram-negative bacteria^9^.

Searching for new sources for the development of novel drugs to treat various cancers is a current subject of public concern. Despite these efforts, cancer remains to be one of the major health problems globally^10^. However, several plant-based compounds are being isolated and screened for their anticancer activity, and fortunately, most of the plant-based compounds are showing amazing results. Evidently, in various in vitro and in vivo studies, it has been found that modified pectin revealed antitumor activity by decreasing adhesion and cell proliferation, as well as induction of apoptosis and migration^9^.

Molecular docking is an exciting and informative approach to understand the interaction between small molecules and biomolecules. It is used to find the active site of the receptor where the small molecules bind or fit. Each computational tool for the docking has an algorithm and gives some physical parameters for understanding the interaction between small molecules binds with the amino-acids available in the receptor or the specific part of protein. The receptors are for a particular purpose and the researchers are trying to find small molecules and their interaction with proteins to study their role as an inhibitor.

The information of the receptors or proteins is available at Research Collaboratory for Structural Bioinformatics (RCSB) as protein data bank (PDB) files with a unique code. For the purpose to study the role of pectin as anti-microbial and anti-cancer, the selected PDB are 1e3g, 3t0c, 5czz, 6j7l, 6v40, 5ibs, 5zsy, and 6ggb. 1e3g represent as a binder to the receptor site of human androgen and progesterone and causes implication in mutations for *E. coli* BL21 (DE3). The different mutations in the receptor are associated with prostate cancer and loss of some sensitivity for the androgen receptors. 3t0c represent the structure of *Streptococcus mutans* MetF and it is complexed with Zinc. It is used to catalyze methyl transfer. The research group reported the crystal structure from the *Streptococcus mutans* at the physiological pH. 5czz represents the crystal structure of *Staphylococcus aureus* Cas9. 6j7l represent structure of *Pseudomonas aeruginosa* Earp in the complex form. It is used for the transfer of rhamnose for the activation. There is a need to find promising inhibitors against the infections that occurred due to this pathogen. 6v40 represents the structure of *Salmonella typhi* TtsA and it is known as a bacterial pathogen. It caused a fever in humans and sometimes, it causes death in infected patients. So, researchers are working to find potential candidates against this bacterium.5ibs encodes the cytoplasmic protein, used for activation of the signaling pathway in humans, used in signaling. The encoded cytoplasmic protein causes somatic mutations and leukemia in mice. Therefore, there is a desperate need to find candidates against this protein. 5zsy, a protein is engaged in the post-transcriptional processing and also in the splicing of the mRNA. Therefore, finding an inhibitor against it is a challenging. 6ggb is about the mutation of cancer, releases unstable protein, and target the chaperons. Hence, it is important to find the inhibitor against it.

**Figure 1:**
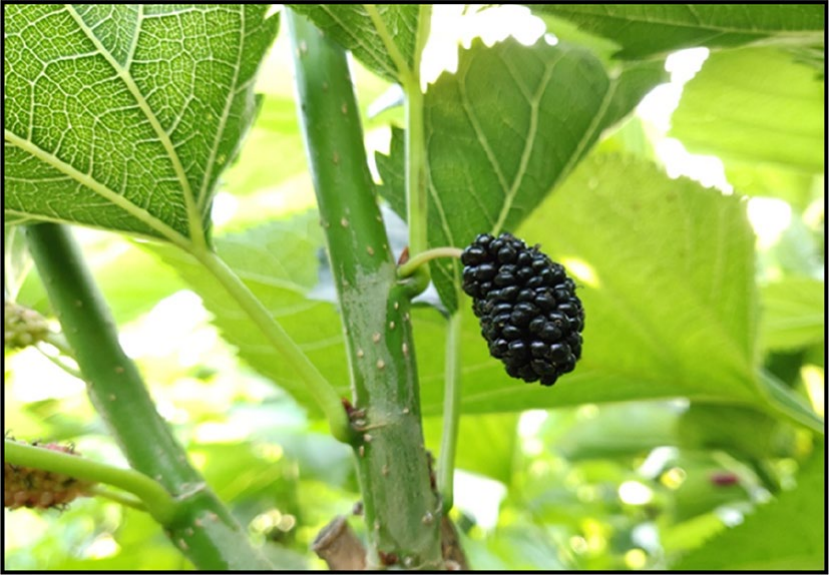
Mulberry fruit: a source of Pectin.

Natural products, mainly citrus fruits, are the significant sources of pectin. However, other fruits, mainly berries, including mulberries, have also been found to be a good source of pectin. Mulberry plant belongs to the family Moraceae, which is widely planted in Asian countries. Mulberries (Fig. 1) are multiple fleshy fruits known as a sorosis, are generally consumed as fresh fruits, jams, and juices. They contain considerable amounts of biologically active ingredients that are associated with potential pharmacological activities and most beneficial for human health^11^. Historically, every vegetative part of mulberry has been effectively used as a traditional medicine in Asia for the treatment of various infectious and chronic diseases, as they are rich source of bioactive compounds.

However, to date, it has not been scientifically well recognized due to the lack of accepted and standardized methodology for its evaluation^12^. To fill this gap, for the first time in the current study, we have targeted to characterize the mulberry fruit pectin of BR-2 and S-13 varieties by ^1^H-NMR spectroscopy and in combination with SEM studies. The antibacterial and anticancer properties of mulberry fruit pectin also evaluated. Further, the molecular docking of pectin is performed to study its biological potency against several bacteria and tumor causing protein.

## Results and discussion

### Extraction of pectin from mulberry fruits

Pectins were extracted from mulberry fruits by acid extraction method, and the percentage of yield was calculated for each extracted pectin samples (S-13 and BR-2) by the following formula:

$$ \begin{aligned} & {\text{Y}}\% = {\text{x}}/{\text{w }}\left( {{1}00} \right) \\ & {\text{BR - 2 variety: }}\,{\text{Y}}\% = { 1}.{4}/{2}0 \, \times { 1}000 \, \times { 1}00 \, = { 7}.0 \\ & {\text{S - 13 variety: }}\,{\text{Y}}\% = \, 0.{95}/{2}0 \, \times { 1}000 \, \times { 1}00 \, = { 4}.{75} \\ \end{aligned} $$

Accordingly, BR-2 and S-13 varieties of mulberry fruit pectins achieved 7.0% and 4.75%, of yield respectively.

### Purification of mulberry fruit pectin

The purification process was conducted by bromine in aqueous solution followed by oxidation, as suggested by Nanji and Chinoy^13^. This method found to be quick and can achieve extreme purity while purification of pectin from crude samples.

### Characterization of mulberry fruit pectin by ^1^H-NMR

**Figure 2:**
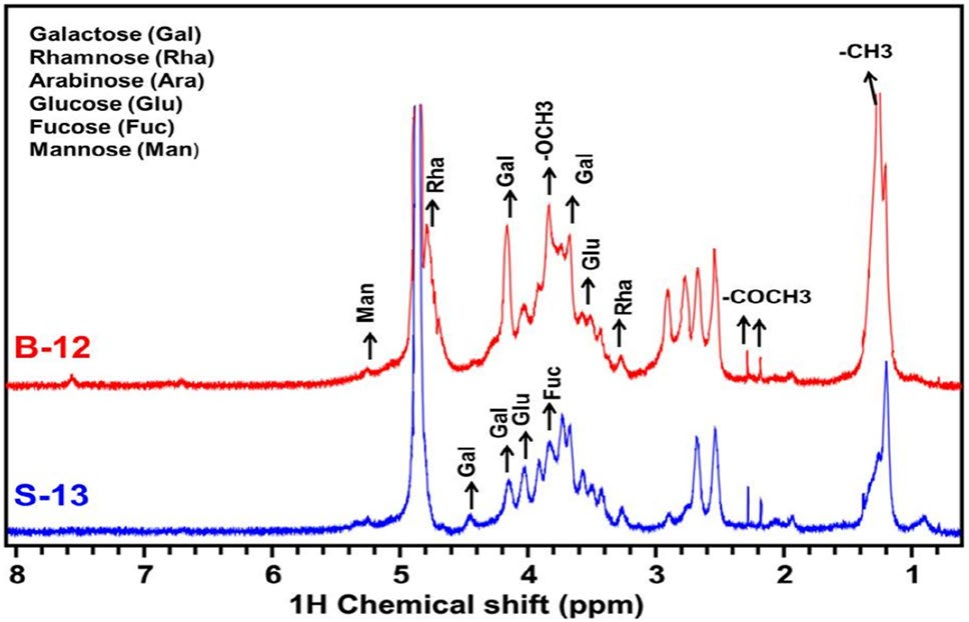
NMR spectra of mulberry Pectin: BR-2 and S-13 varieties.

The stacking of ^1^H-CPMG NMR spectra of pectin samples extracted from BR-2 and S-13 mulberry fruit varieties (Fig. 2).The visual inspection revealed metabolic composition (qualitative) differences between the two pectin types. Further, we performed the quantitative and comparative analysis of concentrations profiles of selected metabolites estimated from the NMR spectra of pectin samples using CHENOMX. As evident from Fig. 2, we were able to identify the presence of six metabolites in different pectin samples, namely: glucose, galactose, rhamnose, arabinose, fucose, and xylose. Similar metabolites have also been identified and quantified previously by Zhi et al. by the pectin samples isolated from citrus fruit through the NMR spectra^14^.

### Anti-bacterial activity of pectin

**Figure 3:**
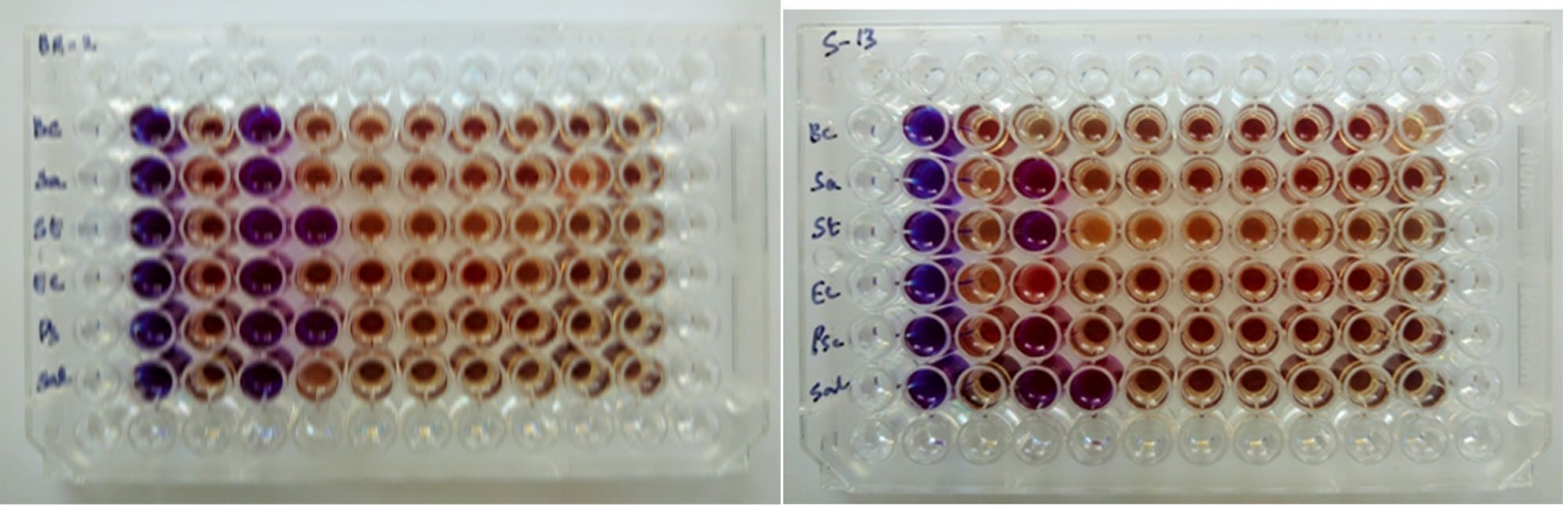
Pectin sample of BR- 2 and S-13 micro titer plates.

Two mulberry fruit pectin varieties, namely BR-2 and S-13 were investigated to evaluate their antibacterial activity against six bacterial strains such as *Bacillus cereus, Staphylococcus aureus*, *Streptococcus mutans, E. coli, Pseudomonas aeruginosa and Salmonella typhi* by MIC method. Evaluation of the antibacterial activity of these pectin varieties recorded in Table 1 and illustrated in Fig. 3 The results revealed that BR-2 mulberry fruit pectin showed higher antibacterial activity against *Streptococcus mutans* (St) (500 µg/ml), which is gram-positive and *Pseudomonas aeruginosa* (Ps) (500 µg/ml) which is gram-negative. Whereas, S-13 mulberry fruit variety revealed higher antibacterial activity against *Salmonella typhi* (Sal)(500 µg/ml), which is gram-negative bacteria. Both the pectin samples (BR-2 and S-13) exhibited antibacterial activity against selected bacterial strains, at a concentration between 500–1000 µg/ml (Table 1). However, the antibacterial activity of S-13 was nil against only two bacterial strains viz. *E. coli* and *Bacillus cereus* (Bc) while they inhibited other pathogens at a concentration of 500–1000 µg/ml (Table 1). Similar results were achieved by Abdel-Massih et al*.*^15^, and Zofou et al*.*^16^, who evaluated the antibacterial activity of pectin against bacteria, Aldana et *al.,* found that edible films prepared with pectin extracts exhibited antibacterial activity^17^. Further, Chaiwarit et al*.*, reported orange oil loaded pectin films possessed antibacterial activity against *Staphylococcus aureus*. Hence, these studies are of a clear indication that pectin could be used as a potential antibacterial drug^18^.

**Table 1:** Minimal inhibitory concentration (MIC) determination of BR-2 and S-13 pectin extracts against bacterial strain.

Mulberry Pectin varieties	Minimum Inhibitory Concentration (µg/ml) of BR-2 and S-13 against selected bacterial strains					
	*Bacillus cereus (Bc) *(µg)	*Staphylococcus aureus (Sa) *(µg)	*Streptococcus mutans (St) *(µg)	*E-coli (Ec) *(µg)	*Pseudomonas aeruginosa (Ps) *(µg)	*Salmonella typhi (Sal) *(µg)
BR-2	1000	1000	500	1000	500	1000
S-13	Nil	1000	1000	Nil	1000	500

### Anti-cancer activity of mulberry fruit pectin

**Figure 4:**
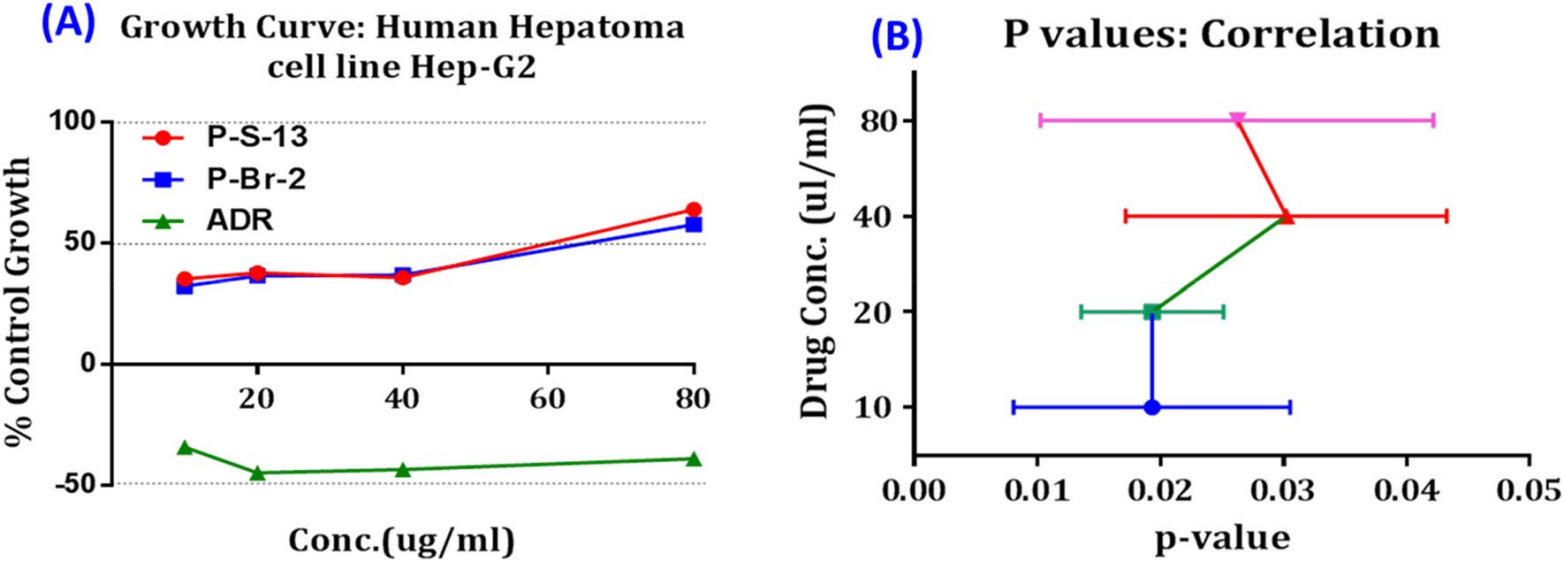
(**a**) Growth curve of Human Hepatoma Cell Line treated with mulberry pectin: BR-2, S-13 and Adriamycin (positive control drug); (**b**) Significant correlation of p-value with different doses of pectin.

**Figure 5:**
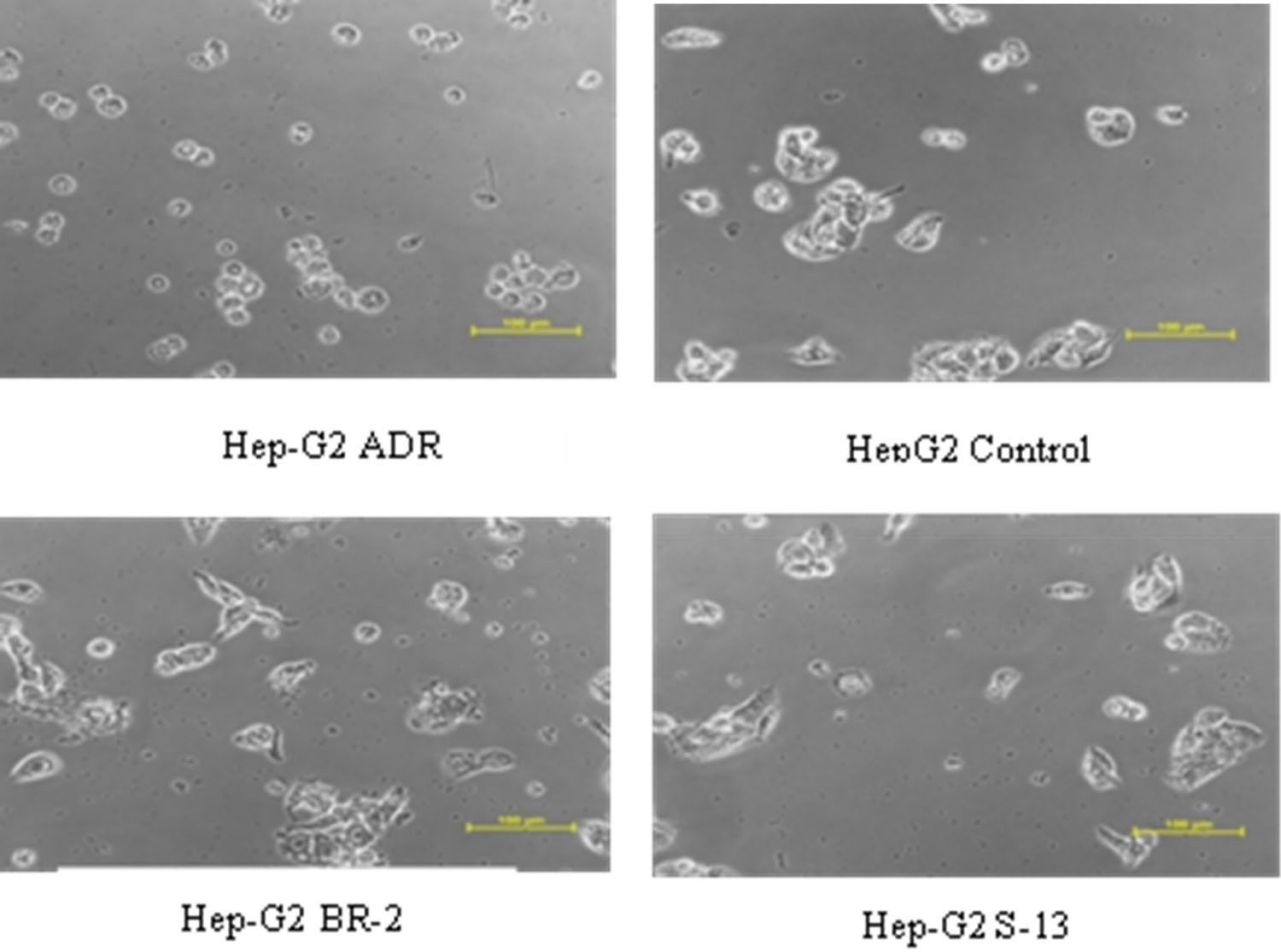
Images of cancer cell line Hep-G2 treated with mulberry pectin BR-2, S-13 and standard drug (Adriamycin).

BR-2 and S-13 mulberry fruit pectin extracts were tested for their anticancer property against the human colon cancer cell line (HT-29) and human hepatoma cell line (HepG2). The extracted pectins inhibited the growth of the human hepatoma cell line (HepG2), eventually leading to apoptosis at the lowest concentration (10 µg/ml). The Mean ± SD of S-13, BR-2, and Adriamycin (ADR) were 43.25 ± 13.94, 40.88 ± 11.47, and -40.35 ± 4.787 respectively. A Staining assay was performed to evaluate the anti-proliferative activity of Pectin extracted from two mulberry fruit varieties such as S-13 and BR-2 against Human Hepatoma Cell lines. According to Fig. 4a, S-13 and BR-2 varieties inhibited the growth of HepG2 cells. Further, it was observed that lower concentrations (10 µg/ml) were more effective in growth inhibition than the higher concentrations (80 µg/ml). The half-inhibitory concentrations (GI50) of Pectin S-13 and BR-2 in HepG2 cells after treatment on an average concentration of 10 μg/mL are revealed 35.3% and 32.3% of inhibition, respectively. Adriamycin being standard anti-cancer drug for cancer chemotherapy significantly inhibited the cell growth of HepG2 cells after treatment at concentration 10 μg/ml. Moreover, pectin at a dose of 80 μg/ml reduces the growth inhibition of HepG2 cells (Fig. 4a). Further confirmation, the growth inhibition potentiality of pectin from S-13 and BR-2 varieties were performed at the dose level 10, 20, 40, and 80 µg/ml against HepG2 cells with p-value correlation. Interestingly, all doses were significantly correlated with each other (Fig. 4b). Taken together, these results suggest that pectin exhibits a potent anti-proliferative effect on HCC cells. It has also been observed that both BR-2 and S-13 mulberry fruit pectins are not active against the human colon cancer cell lines (HT-29). However, both the pectin samples found to be active against the human hepatoma cell line of HepG2 at lower concentrations (Table 2, Figs. 4, 5). Similar compatibility studies conducted by Leclere et al*.,* revealed that the citrus pectin modified by heat treatment-induced cell death in HepG2^19^. Our results are also in accordance with those of Gupta and Kumar, who studied the efficacy of drug-loaded pectin-based nanoparticles against HepG2 hepatoma cells. Further, pectin loaded nanoparticles actively targeted the hepatoma cells^20^. The cytotoxicity studies of pectin-naringenin conjugate showed anti-cancer activity against NIH: OVCAR-5 cells^21^. Ogutu *et al.,* observed the apoptosis effect of Ultrasonic Modified Sweet Potato Pectin in Colon Cancer (HT-29) Cell Line^22^. The above studies describe the cytotoxic activity of pectin, which could exploit to identify active compounds against human hepatoma cancer cells for further studies.

**Table 2:** Growth inhibition percentage at different dose concentrations of pectin on human hepatoma cell line (Hep-G2).

Drugs	Drug Concentrations (10, 20, 40 and 80 µg/ml)															
	Experiment 1 (µg/ml)				Experiment 2 (µg/ml)				Experiment 3 (µg/ml)				Average Values (µg/ml)			
	10	20	40	80	10	20	40	80	10	20	40	80	10	20	40	80
	Percentage of Control Growth = 100															
S-13	34.2	44.1	38.2	67.5	34.4	36.8	34.5	55.4	37.4	32.4	34.8	69.3	35.3	37.8	35.8	64.1
BR-2	40.8	41.4	28.7	58.4	24.1	29.8	44.0	53.1	32.0	38.5	37.9	62.0	32.3	36.5	36.9	57.8
Adriam-ycin(ADR)	− 34.2	− 44.8	− 43.4	− 39.0	− 34.2	− 44.8	− 43.4	− 39.0	− 34.2	− 44.8	− 43.4	− 39.0	− 34.2	− 44.8	− 43.4	− 39.0

### Surface analysis of pectin Using Scanning electron Microscope

**Figure 6:**
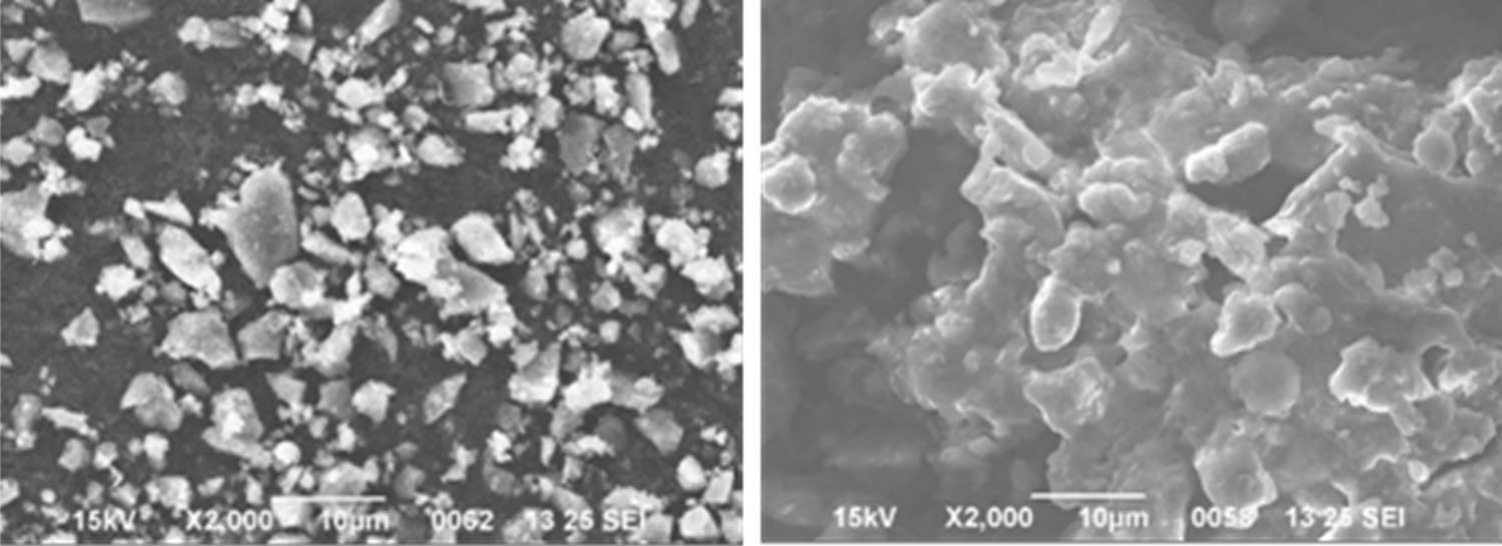
Scanning electron microscope images of S-13 (Left) and BR-2 (Right) verities of mulberry fruit pectin at 2000 × magnification.

A small uneven stone-like SEM structure obtained from the S-13 pectin variety, whereas; BR-2 pectin shows clustered structure (Fig. 6) A distinct image of pectin was obtained from mangosteen peel extract^6^, Dranca and Oroian observe closely packed and less smooth structure of the pectin obtained from *Malus domestica* ‘Falticeni’ apple^23^, and Fracasso et al*.* also found a fibrous and rough structure, with mean sizes between 5 and 100 µm of citrus pectin using SEM^24^. All these SEM studies are contradictory to the current morphology of mulberry fruit pectin. SEM micrographs of BR-2 and S-13 pectin powder revealed a rough rocky surface with irregular shapes (Fig. 6).

### Molecular Docking of mulberry fruit pectin

**Figure 7:**
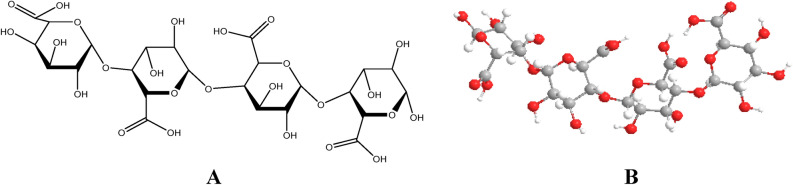
Structure of the pectin (tetramer) taken in 2D and 3D.

For the molecular docking, the structure of pectin is drawn as in Fig. 7 and optimized using Gaussian. Further, the PDB files (1e3g, 3t0c, 5czz, 6j7l, 6v40, 5ibs, 5zsy, and 6ggb) were taken from the RCSB and prepared for the docking using chimera. Herein, removal of ligands/ cofactors, if any, the addition of atoms is done to get good results from to docking to study the interaction between the pectin and desired receptors of the pectin with the receptors of the PDBs (1e3g, 3t0c, 5czz, 6j7l, 6v40, 5ibs, 5zsy, and 6ggb) was performed using the iGEMDOCK and the interaction was studied in the form of physical data that is binding energy as in Table 3^25^.

**Table 3:** Interaction of the receptors of the PDBs (1e3g, 3t0c, 5czz, 6j7l, 6v40, 5ibs, 5zsy and 6ggb) with the pectin.

PDB ID	Total Energy	VDW	HBond	Elec
1e3g	− 145.085	− 93.0408	− 49.1681	− 2.87612
3t0c	− 163.702	− 118.4	− 45.997	0.694842
5czz	− 156.107	− 92.0455	− 49.2918	− 14.7692
6j7l	− 150.364	− 94.7174	− 54.6745	− 0.972
6v40	− 166.859	− 105.112	− 59.3093	− 2.43757
5ibs	− 141.709	− 96.4091	− 45.4423	0.142841
5zsy	− 121.405	− 81.2714	− 34.8403	− 5.29285
6ggb	− 121.405	− 81.2714	− 34.8403	− 5.29285

**Figure 8:**
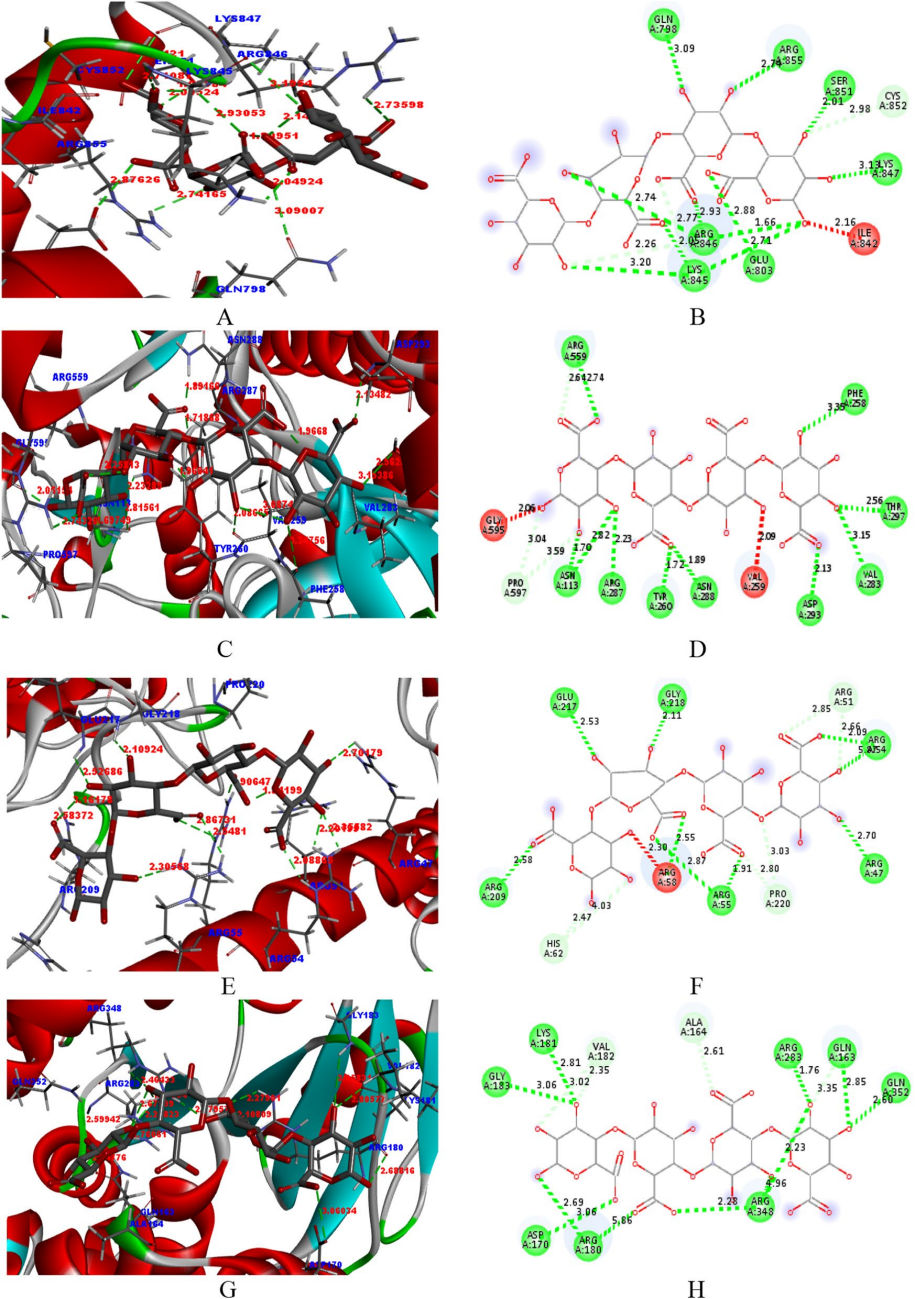

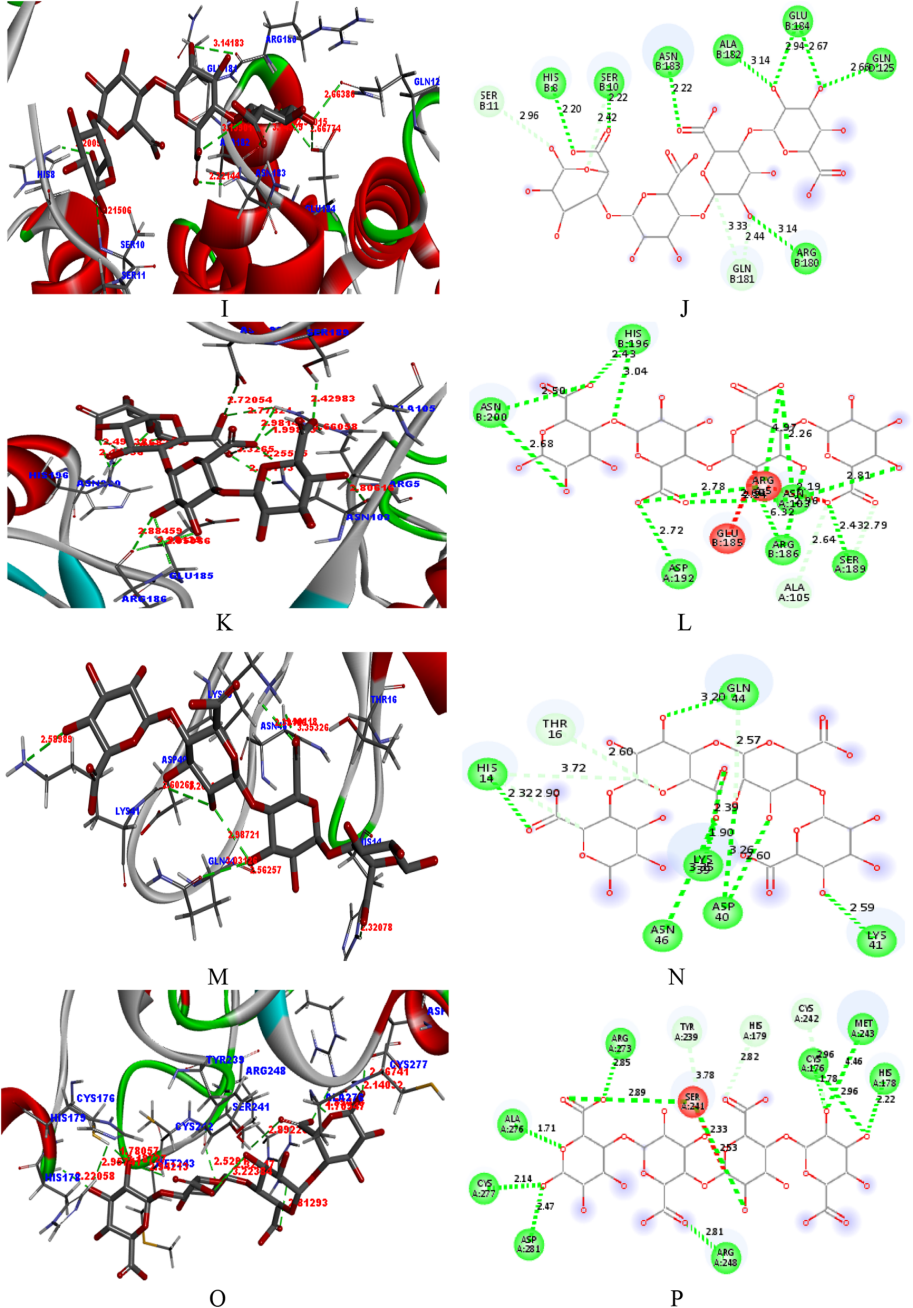
2D- and 3D- views for the interaction of pectin with the receptors (1e3g, 3t0c, 5czz, 6j7l, 6v40, 5ibs, 5zsy and 6ggb).

Herein, it is clearly visible that the pectin binds effectively with the receptors of the above mentioned PDBs. Further, the interaction between the pectin and the receptors were visualized using computational tools as in Fig. 8. The docking posed (3D) for the interaction was taken and seen the effective binding in cavity of the receptor. Further, the interaction of the pectin with amino-acids of the receptors can be seen from the 2-dimensional views.

Hydrogen bonding and other interactions between the pectin and the receptors can be analyzed with the help of Table 4. Herein, hydrogen bonding (classical and non-classical) and miscellaneous interaction are given. It is considered that more the number of a hydrogen bond between the ligand and the receptors indicate more effective binding. Therefore, pectin can be considered a potential inhibitor for the said species. From Table 4, it can be seen that pectin binds with the receptor in the PDBs (1e3g, 3t0c, 5czz, 6j7l, 6v40, 5ibs, 5zsy, and 6ggb). Pectin forms hydrogen bonds with the amino-acids of the active cavity of the receptor.

**Table 4:** Interactions (hydrogen bonding and others) of pectin with different PDBs.

PDB	H-bonds				Miscellaneous with their distance (Å)	
	Classical with their distance (Å)		Non-classical with their distance (Å)			
1E3G	GLN789	3.09	CYS852	2.98		
	ARG855	2.74	ARG846	2.26		
	SER851	2.01	LYS845	2.77		
	LYS847	3.13				
	GLU803	2.88				
	LYS845	2.71, 3.20, 2.05				
	ARG846	1.66, 2.74, 2.93				
3T0C	ARG559	2.74	PRO597	3.04, 3.59	GLY595	2.06
	PHE258	3.35	ARG559	2.64	VAL259	2.09
	THR267	2.56				
	VAL283	3.15				
	ASP293	2.13				
	ASN288	1.89				
	TYR260	1.72				
	ARG287	2.23				
	ASN113	1.70, 2.82				
5CZZ	GLU217	2.53	ARG51	2.85, 2.66	ARG56	2.30
	GLY218	2.11	PRO220	3.03, 2.80		
	ARG54	2.09, 2.35	HIS62	2.47, 4.03		
	ARG47	2.70				
	ARG55	1.91, 2.87				
	ARG58	2.55				
	ARG209	2.58				
6J7L	GLU183	3.06	VAL182	2.35, 3.02		
	LYS181	2.81	ALA164	2.61		
	ARG283	1.76	GLN163	3.35		
	GLN163	2.85				
	GLN352	2.60				
	ARG348	4.96, 2.28				
	ARG180	5.86, 2.69				
	ASP170	3.06				
6V40	HIS8	2.20	SER11	2.96		
	SER10	2.20	SER10	2.42		
	ASN183	2.22	GLN181	3.33, 2.44		
	ALA182	3.14				
	GLU184	2.94, 2.67				
	GLN125	2.66				
	ARG180	3.14				
5IBS	ASN200	2.68, 2.50	GLU185	2.24,	GLU185	2.24
	HIS196	2.43, 3.04	HIS196	2.65	ARG5	1.66, 2.19
	ASP192	2.72	ALA105	2.64		
	SER189	2.64	SER189	2.79, 2.64		
	ARG5	2.78, 2.98, 1.66				
	ASN103	2.81, 2.23, 2.60				
	GLU186	2.96, 2.88, 2.98				
5ZSY	HIS14	2.32	GLN44	2.57		
	GLN44	3.03, 2.56	THR16	2.60		
	LYS41	2.59	HIS14	3.72, 2.90		
	ASP40	3.26, 2.60				
	LYS39	1.90, 2.39				
	ASN46	3.35				
6GGB	ALA276	1.71	SER241	2.75	SER241	2.33

Pectin interacts with different amino-acids present at the receptor site of 1E3G. It forms classical hydrogen bonds with the GLN789, ARG855, SER851, LYS847, GLU803, LYS845 and ARG846 and non-classical hydrogen bonds with CYS852, ARG846 and LYS845. Further, no other interactions are observed. Pectin interacts with different amino-acids present at the receptor site of 3T0C. It forms classical hydrogen bonds with the ARG559, PHE258, THR267, VAL283, ASP293, ASN288, TYR260, ARG287 and ASN113 and non-classical hydrogen bonds with PRO597 and ARG559. Further, some other interactions are observed with GLY595 and VAL259. Pectin interacts with different amino-acids present at the receptor site of CZZ. It forms classical hydrogen bonds with the GLU217, GLY218, ARG54, ARG47, ARG55, ARG58 and ARG209 and non-classical hydrogen bonds with ARG51, PRO220, and HIS62. Further, some other interactions are observed with ARG56. Pectin interacts with different amino-acids present at the receptor site of 6J7L. It forms classical hydrogen bonds with the GLU183, LYS181, ARG283, GLN163, GLN352, ARG348, ARG180 and ASP170 and non-classical hydrogen bonds with VAL182, ALA164, and HIS62. Further, no interactions are observed. Pectin interacts with different amino-acids present at the receptor site of 6V40. It forms classical hydrogen bonds with the HIS8, SER10, ASN183, ALA182, GLU184, GLN125, and ARG180 while forms non-classical hydrogen bonds with SER11, SER10, and GLN181. Further, no other interactions are observed. Pectin interacts with different amino-acids present at the receptor site of 5IBS. It forms classical hydrogen bonds with the ASN200, HIS196, ASP192, SER189, ARG5, ASN103, and GLU186 while forms non-classical hydrogen bonds with GLU185, HIS196, ALA105, and SER189. Further, some other interactions are observed with GLU185 and ARG5. Pectin interacts with different amino-acids present at the receptor site of 5ZSY. It forms classical hydrogen bonds with the HIS14, GLN44, LYS41, ASP40, LYS39, and ASN46 while forms non-classical hydrogen bonds with GLN44, THR16, and HIS14. Further, no other interactions are observed. Pectin interacts with different amino-acids present at the receptor site of 6GGB. It forms classical hydrogen bonds with the ALA276 and forms non-classical hydrogen bonds with SER241. Further, some other interactions are observed with SER241.

**Figure 9:**
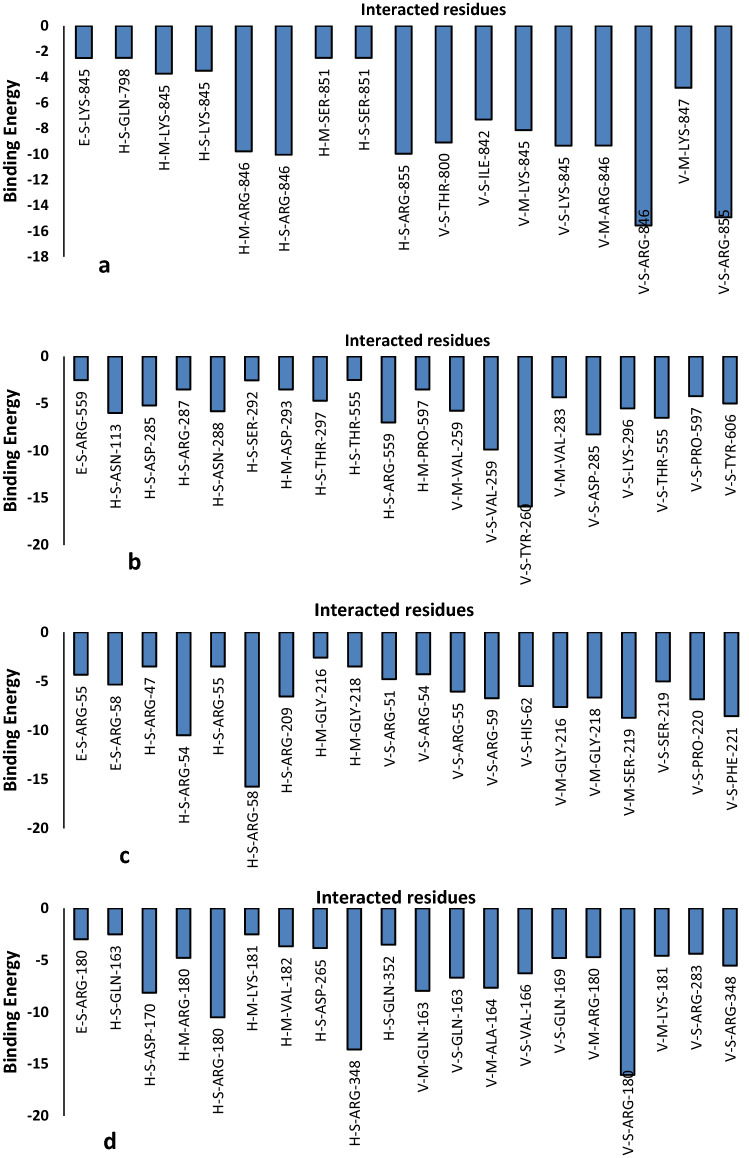

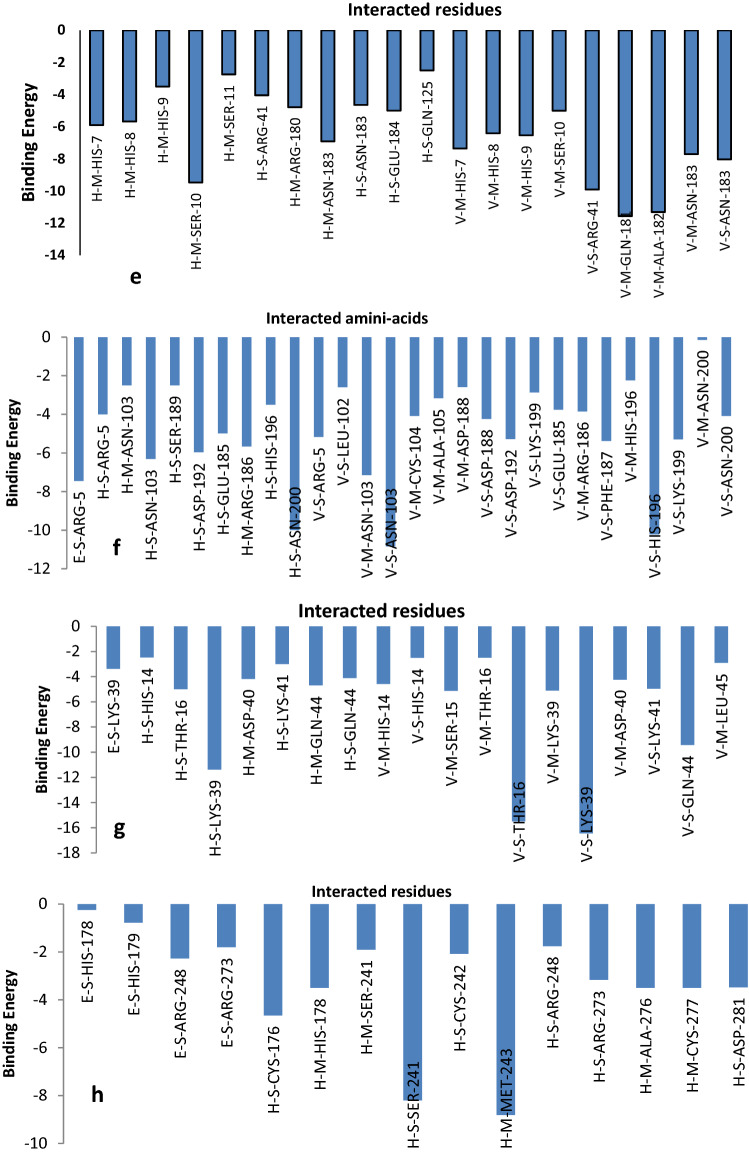
Plots of the amino acids significant interacted with pectin v/s energy contributed.

Based on Fig. 9, it is understood that pectin interacts with some of the amino-acids of the receptors significantly. Pectin interacts with the amino-acids of 1CEG in terms of their energy and it has been observed that ARG-855 and ARG-846 contribute significantly. Pectin interacts with the amino-acids of 3TOC in terms of their energy and it has been observed that TYP-260 contributes significantly. Pectin interacts with the amino-acids of 5CZZ in terms of their energy and it has been observed that ARG-54 and ARG-58 contributes significantly. Pectin interacts with the amino-acids of 6J7L in terms of their energy and it has been observed that ARG-180 and ARG-348 contributes significantly. Pectin interacts with the amino-acids of 6V40 in terms of their energy and it has been observed that SER-10, ARG-41, GLN-181 and ALA-182 contribute significantly. Pectin interacts with the amino-acids of 5ibs in terms of their energy and it has been observed that ARG-5, ASN-103, ASN-200, HIS-196 contributes significantly. Pectin interacts with the amino-acids of 5ZSY in terms of their energy and it has been observed that LYS-39, THR-16, LYS-39 and GLN-44 contributes significantly. Pectin interacts with the amino-acids of 6GGB in terms of their energy and it has been observed that SER-241, MET-243, CYS-176 and HIS-178contribute significantly.

## Materials and methods

Ripened mulberry fruit varieties namely BR2 and S13 were collected from Mulberry garden of the Department of Zoology, Babasaheb Bhimrao Ambedkar University, Lucknow, India. After the collection of fresh mulberry, fruits were washed and dried in a hot air oven at 55 ͦ C until the constant weight was achieved. Dried fruits were crushed into a fine powder and kept in polyethylene bags and stored at − 15 °C for the extraction of pectin.

### Extraction of pectin

The extraction of pectin was carried out in Citric acid (pH 2.2) at 70 °C. 400 mL of extraction medium was prepared consist of acid and water. 20 g of mulberry fruit powder of BR-2 and S-13 was added in the extraction medium separately and kept at 100 ͦ C temperature for 80 min for the extraction of pectin. Later on, the mixture was filtered with the help of filter paper and the supernatant was discarded and equal the volume of 95% ethanol was added to the resultant filtrate containing the pectin. When the pectin started precipitating, the filtrate was subjected to centrifuge at 10,000 rpm for 10 min, after that pellet was recovered after washing by 70% ethanol and dried in a hot air oven at 50^0^C. Resultant dried pectin was converted into a fine powder and stored in an airtight Eppendorf tube for further studies. The percentage yield was calculated for each extracted pectin sample by the following formula^26^:

$$ {\text{Y}}\% \, = {\text{ x}}/{\text{w }}\left( {{1}00} \right) $$

where Y = Pectin yield, x = Weight of dried pectin extracted, w = Weight of fine mulberry powder taken from the extraction.

### Purification of pectin

Pectin of both the selected mulberry varieties was mixed with water and boiled to make a solution separately. 20 ml of N/10 bromine water was added to 50 ml of the cooled pectin solution. Oxidation was allowed to proceed for varying periods at different temperatures. Excess bromine water was removed by shaking 2–3 times and added ether until the aqueous layer was colorless. Pectin samples were then precipitated by 95% alcohol and after 2 h were filtered through a muslin cloth. Filtered pectins were finally passed through various grades of alcohol and dried in an oven at 100 °C for 20 h as described by the researchers^13^.

### Characterization of pectin by ^1^H^-^NMR

NMR sample preparation, data collection, and spectral data processing were performed following the procedure described previously^4,14,27^. Initially, the dried pectin powder sample was dissolved in 700µL of NMR buffer (100 mM sodium phosphate buffer of pH 7.3 prepared in 100% deuterated water containing 0.9% NaCl, 0.1% sodium azide, and 1 mM TMSP (i.e. 3-(trimethylsilyl) propionic-2,2,3,3-d_4_ acid as internal standard). The resulted solution sample was then vortex-mixed and centrifuged to remove any remaining undissolved material. From each sample, 600µL of the supernatant was transferred into a 5 mm NMR tube. The NMR experiments were recorded on an 800 MHz NMR spectrometer (Bruker-BioSpinAvance III, Bruker Corporation, Silberstreifen 4, 76,287 Rheinstetten, Germany) equipped with 18.79 T standard bore magnet, 5-mm triple resonance inverse TCI CryoProbe (TCI ^1^H-^13^C/^15^ N-^2^H + XYZ gradients). All spectra were recorded at 298 K using CPMG (Carr-Purcell- Meiboom-Gill) pulse sequence (cpmgpr1d, standard Bruker pulse program) with pre-saturation of water during the recycle delay. This resulted in a one-dimensional ^1^H NMR spectrum for each sample (BR-2 and S-13) and used for the quantitative profiling of pectin metabolic entities. The various acquisition parameters used for recording each 1D ^1^H CPMG NMR spectrum was: spectral sweep width: 20 ppm; data point: 64 K; the flip angle of radiofrequency pulse: 90ͦ; total relaxation delay (RD): 5 s; a number of scans: 128; window function: exponential; line broadening: 0.3 Hz. All the spectra were processed using Topspin (v2.1, Bruker Biospin NMR data processing software) and chemical shifts, δ were expressed in ppm and referenced to TSP methyl protons at 0.0 ppm as an internal reference standard. For quantitative profiling, we used commercial software CHENOMX (v8.2, Edmonton, Canada). Each the NMR spectrum is first opened into the PROCESSOR module of CHENOMX for manual phase and baseline correction and re-calibrated to TSP methyl proton signal at 0.0 ppm. The resulted spectra were then imported into the PROFILER-Module of CHENOMX and concentrations of selected metabolites (i.e. galactose, rhamnose, arabinose, fucose, and xylose) were estimated for all the pectin samples.

### Antibacterial activity of mulberry pectin

Antibacterial activity of mulberry pectin was assessed by the resazurin assay method utilizing microtitre-plate^28^.

#### Micro titer plate method (96 wells) to check the minimum inhibition concentration (MIC)

The Pectin sample (BR-2 and S-13) were assessed for their MIC against 6 bacterial strains namely, *Bacillus cereus, Staphylococcus aureus, Streptococcus mutans, E-coli, Pseudomonas aeruginosa,* and *Salmonella typhi* .

***Sample preparation:*** 10 mg of the sample was dissolved in 1 mL Dimethyl sulfoxide (DMSO) and 100 µL of dissolved samples were used to check the Minimum Inhibition Concentration (MIC) for respective organisms.

***Media preparation***: Luria Bertani (LB) broth (tryptone 10 g, sodium chloride 10 g, yeast extract 6 g, and distilled water 1000 mL). 100 mL of LB broth was prepared and autoclaved at 121 °C for 15 min.

***Plate preparation***: 300 µL deionized water was added in the wells of microtiter plates (A_1_ to A_12_, B_12_ to H_12_, H_11_ to H_1_ and G_1_ to B_1_) to prevent the sample from drying. 100 µL sterilized LB broth was added to all the remaining wells. 100 µL of 0.1% resazurin dye in wells B_2_ to G_2_ was added as color blank. In wells B_3_ to G_3_ test organisms and 100 µL of 0.1% resazurin dye were added as culture control. 100 µL of the respective sample added in wells B_4_ to F_4_ and serially diluted by transferring 100 µL of the mixture to subsequent wells in respective plates up to B_11_ to F_11_ and 100 µL of the excess sample was discarded from 11th well respectively, 100 µL of the test organisms and 100 µL of 0.1% of resazurin dye was added to the diluted samples. The plates were incubated at 37 °C for 24 h. The presence of blue color indicates no growth of the organism (inhibited).

***Pectin sample BR-2 and S-13 to test against Bacteria***: 100 µL of culture was inoculated to serially diluted sample wells (*Bacillus cereus* was inoculated to wells from B_4_ to B_11_, *Staphylococcus aureus was* inoculated to wells from C_4_ to C_11_, *Streptococcus mutans* was inoculated to wells from D_4_ to D_11_, *E-coli* was inoculated to wells from E_4_ to E_11_, *Pseudomonas aeruginosa* was inoculated to wells from F_4_ to F_11_ and *Salmonella typhi was* inoculated to wells from G_4_ to G_11_) and 100 µL of 0.1% of resazurin dye was added to all the test sample wells. The plates were incubated at 37 °C for 24 h.

### Anticancer property of mulberry fruit pectin

The anticancer activity of pectin samples were evaluated using SRB protocol assay as described by Pandey et al.^29^, and Elankani et al.^30^. The cell lines (HT-29 and Hep-G2) were grown in RPMI 1640 medium containing 10% fetal bovine serum and 2 mM L-glutamine. Considering the current screening experiment, cells were inoculated into 96 well microtiter plates in 100 µL at plating densities as shown in the study and depending on the doubling time of individual cell lines. After cell inoculation, the microtiter plates were incubated at 37° C, 5% CO_2_, 95% air, and 100% relative humidity for 24 h prior to the addition of experimental drugs. Experimental drugs were solubilized in an appropriate solvent at 100 mg/ml and diluted to 1 mg/ml using water and stored frozen prior to use. At the time of drug addition, an aliquot of frozen concentrate (1 mg/ml) was thawed and diluted to 100 μg/ml, 200 μg/ml, 400 μg/ml and 800 μg/ml with complete medium containing test article. Aliquots of 10 µl of these different drug dilutions were added to the appropriate microtiter wells already containing 90 µl of the medium, resulting in the required final drug concentrations i.e.10 μg/ml, 20 μg/ml, 40 μg/ml, 80 μg/ml. After compound addition, plates were incubated at standard conditions for 48 h and the assay was terminated by the addition of cold TCA. Cells were fixed in situ by the gentle addition of 50 µl of cold 30% (w/v) TCA (final concentration, 10% TCA) and incubated for 60 min at 4 °C. The supernatant was discarded; the plates were washed five times with tap water and air-dried. Sulforhodamine B (SRB) solution (50 µl) at 0.4% (w/v) in 1% acetic acid was added to each of the wells, and plates were incubated for 20 min at room temperature. After staining, the unbound dye was recovered and the residual dye was removed by washing five times with 1% acetic acid. The plates were air-dried. The bound stain was subsequently eluted with 10 mM trizma base and the absorbance was read on a plate reader at a wavelength of 540 nm with 690 nm reference wavelength. Percentage of growth was calculated on a plate-by-plate basis for test wells relative to control wells and expressed as the ratio of average absorbance of the test wells to the average absorbance of the control wells * 100. Further, using the six absorbance measurements time zero (Tz), control growth (C), and test growth in the presence of drug at the four concentration levels (Ti)], the percentage of growth was calculated at each of the drug concentration levels. Percentage growth inhibition was calculated as:

$$ \left[ {{\text{Ti}}/{\text{C}}} \right] \, \times { 1}00 \, \% $$

### Morphological characteristics of mulberry fruit Pectin

The SEM was applied to elucidate morphological features of mulberry pectin samples which were extracted by acidic extraction method. The pectin samples were dried in the microwave at 100 °C and mounted with double-sided carbon tape on aluminum stubs, sputter-coated with palladium coater (Auto fine coater JFC- 1600 JEOL, Japan). Both selected pectin samples were observed by JEOL, JSM 6490 LV (Tokyo, Japan) under high vacuum scanning electron microscope at different magnification, as described (24).

#### Preparation of the receptors for different purposes and pectin (tetramer)

Pectin is a polymer and proposes to be effective against the microbes as well as cancer cells^31,32^. Therefore, to corroborate the experimental work, theoretical studies i.e. molecular docking has been performed. Herein, the tetramer unit of the pectin was drawn as in Fig. 7 and studied its interaction with the PDB (1e3g, 3t0c, 5czz, 6j7l, and 6v40) used to explore the potential of the pectin^33–40^.

### Molecular docking between pectin and different receptors

In the present work, an item dock was used for the molecular docking between the pectin and different receptors. The docking of the pectin with the receptor gives three solutions and each of the solutions is based on seventy generations^33–48^. The total binding energy for the interaction between the pectin and active amino-acids of the receptors is determined based on the energy contributed by the hydrogen bonding, van der Waals, and electrostatic interaction^49–57^. Herein, the PDBs for the different purposes are taken from the RCSB (https://www.rcsb.org). The PDBs are prepared for the purpose of molecular docking using computational tools like Chimera 1.11.2. It is used for the removal of the ligands present, the addition of the deficit hydrogen and charges by using AMBER.ff14SB force field via the dock prep module. Further, the structure of the pectin was geometrically optimized for docking using the computational tools, Gaussian 9.0. It should be done to make the molecule free from the steric clashes. Post dock modeling or the analysis is important and used to get information in the form of physical data and pictorial view for interaction between the pectin and the receptor or target. Herein, two as well three-dimensional views can be collected and analysed using BIOVIA Discovery Studio^58–62^.

## Conclusion

There is an emergent need to find out complementary and safe treatment strategies based on bioactive natural products against chronic diseases, due to emerging resistance against the majority of existing antibiotics. The current study highlighted that mulberry fruit pectin extracted from BR-2 and S-13 varieties showed a significant level of antibacterial activity against all tested bacterial strains and cytotoxic properties on the HepG2 cell line. Further, the tetramer has been taken as the basic unit for pectin. Based on molecular interaction through docking, pectin binds effectively with the receptors (1e3g, 3t0c, 5czz, 6j7l, 6v40, 5ibs, 5zsy, and 6ggb). This scenario describes the pectin compounds isolated from mulberries have great biological activities with cellular level target abilities with proven a promising anti-microbial and anti-cancer substances of natural products. Further, re-engineering of these pectins will certainly offer interesting and immense future impact with unexplored biological activities. Accordingly, leading to compass for exploring the mechanism of action through in vivo and clinical studies.
